# Early childhood teachers’ professional learning about ICT implementation in kindergarten curriculum: A qualitative exploratory study in China

**DOI:** 10.3389/fpsyg.2022.1008372

**Published:** 2022-10-21

**Authors:** Tian Yang, Xiumin Hong

**Affiliations:** ^1^Center for Educational Science and Technology, Beijing Normal University, Beijing, China; ^2^Faculty of Education and Social Work, The University of Auckland, Auckland, New Zealand; ^3^Institute of Early Childhood Education, Faculty of Education, Beijing Normal University, Beijing, China

**Keywords:** early childhood teachers, professional learning, professional development, TPACK, China, kindergarten curriculum, preschool education

## Abstract

Many teachers have begun to adopt information and communication technologies (ICT) in early childhood education (ECE) settings to support children’s learning. However, research shows that ECE teachers’ ICT implementation practice is not always appropriate, and their limited professional learning opportunities is one reason for this. Researchers worldwide have called for more understanding of professional learning that supports ECE teachers’ use of ICT in the kindergarten curriculum. In China, although ECE teachers’ ICT competencies and skills are required in national documents, little has been reported about how they are supported in learning about implementing ICT in the current curriculum. Drawing on the Technological Pedagogical Content Knowledge (TPACK) model, this study investigates how a small group of ECE teachers in China have experienced professional learning regarding ICT-related curriculum. By conducting individual interviews and analyzing public and teachers’ personal documents, this study finds that teacher participants had received diverse types of professional learning opportunities related to ICT implementation. These opportunities were provided by different organizations; however, one shared feature was a focus on the technical level of how to use ICT devices. This study also discusses the teaching-research culture underpinning participants’ professional learning. This article provides significant implications for advancing current professional learning programs.

## Introduction

There is ample empirical evidence showing that information and communication technologies (ICT) are implemented in early childhood education (ECE) settings all over the world (Gibbons, 2010; Dong, 2014). Correspondingly, the ICT-supported curriculum has appeared in some ECE settings, as teachers implement ICT to transform practices to help children gain more learning and play experiences, and their ultimate goal is to support children’s learning in this digital era (Nikolopoulou and Gialamas, 2015; Mertala, 2019).

However, previous researchers have indicated that training programs related to ICT-related curriculum are insufficient and inefficient for ECE teachers (Nikolopoulou and Gialamas, 2015; Blackwell et al., 2016), which might prevent teachers from appropriately using ICT in their kindergarten curriculum. Researchers in China have reported similar findings (Dong, 2014; Liu, 2017), but they seemed to provide overall descriptions without giving detailed explanations about the types and forms of professional learning opportunities that China’s ECE teachers experienced.

Thus, research needs to unpack more specifically what types of professional learning opportunities teachers have had and how those professional learning opportunities support ICT-related curriculum in kindergartens. This study aims to investigate the ways in which ECE teachers in China have experienced those professional learning opportunities. Obtaining such understanding is timely and important, as the literature on this research topic has been focused on Western societies, and the Asian and Chinese contexts remain under-researched. The findings will provide empirical evidence and highlight issues existing in teachers’ professional development regarding ICT implementation, which can contribute to the current knowledge base in this field and shed light on future professional development programs and policy-making.

### ICT implementation by ECE teachers

The importance of teachers developing ICT expertise has been noted by both international and national researchers (Dong, 2014; Blackwell et al., 2016; Johnston et al., 2020). Although the role that ICT plays in children’s development is still debated, researchers have generally agreed that the current focus should be related to the question of *in what ways* should ICT be used to maximize its benefits, rather than *whether* ICT affects children’s development (Blackwell et al., 2016; Bird, 2018). Ihmeideh and Al-Maadadi (2018) also have emphasized that the responsibility of integrating ICT into ECE settings falls on the shoulders of teachers. Therefore, the question how children use ICT with the guidance and support of teachers’ is important for ECE settings. However, there is ample evidence showing that ECE teachers implement ICT for children in an inappropriate manner, such as providing insufficient responsive guidance and limiting children’s interactions with ICT (Dong, 2014; Sargent, 2017). Inappropriate ICT implementation may cause children to have negative feelings and limited learning opportunities, which may have a long-term effect on children’s ICT-supported learning (Dong, 2014; Park, 2015). Given that teachers are the ones who decide how children experience ICT in ECE settings, their professional learning experience regarding ICT-related curriculum is an important research topic.

Researchers have drawn teachers’ attention to teachers’ professional development as it relates to ICT competence, identifying teacher training as the main factor influencing their practice (Blackwell et al., 2016; Sargent, 2017; Dardanou and Kofoed, 2019). For instance, in Norway, Dardanou and Kofoed (2019) have reported that teachers lack knowledge about how to deal with ethical issues regarding ICT implementation due to the absence of relevant training, in Jordan, Ihmeideh (2009) found that ECE teachers lack funding to participate in ICT-related training and therefore stick to old approaches to teaching, and in Greece, Nikolopoulou and Gialamas (2015) reported that ECE teachers had more training opportunities on the technical level than the pedagogical level and indicated that their previous training did not enhance their confidence with ICT implementation. However, previous researchers simply described teachers’ ICT-related training without specifically investigating the ways in which they experienced those training opportunities.

Research shows that ECE teachers’ training experience in ICT implementation can effectively enhance teachers’ motivation to ICT use and develop knowledge about how to support children’s learning in appropriate ways. Ihmeideh and Al-Maadadi (2018) provided an intervention training program (that focused on teachers’ understanding of ICT itself, ICT-related teaching strategies, and examples of effective ICT implementation practices) for ECE teachers in Qatar, and found that teachers tended to experience a careful decision-making process when about to implement ICT in learning activities after the training. In Australia, after examining a professional learning approach (i.e., practitioner inquiry) adopted to facilitate ICT implementation in ECE settings, Johnston et al. (2020) reported the value of individual and collaborative group reflections and the significance of various professional learning resources (such as professional reading, group discussion and workshops). However, at present, the number of studies on how ECE teachers experience ICT-related professional development opportunities is still limited.

Albeit little empirical evidence regarding ECE teachers’ professional learning experience relating to ICT implementation exists, researchers have provided suggestions to support ECE teachers’ practices, such as emphasizing that training programs should be carefully designed to provide teachers with skills to evaluate appropriate devices or software (Nikolopoulou and Gialamas, 2015). Mertala (2019) suggested that ECE teachers should have more training opportunities that go beyond the focus on using ICT for teaching curricular subjects. Furthermore, Sargent (2017) advocated that ongoing support could help teachers experience sustainable changes in knowledge and skills related to ICT implementation. Researchers have argued that ECE teachers’ observation and reflection on ICT use are important parts of professional development because the process of “observation, reflection, changes to practice and more reflection” (Vidal-Hall et al., 2020, p. 176) can result in a modification of view, and therefore, a transformation in teaching practice, which finally influences children’s learning experience. These studies are important for understanding how ECE teachers could be provided with professional learning opportunities to support their ICT implementation.

In summary, there have been more and more calls for a focus on ECE teachers’ professional learning in relation to ICT implementation in the curriculum, as the ICT-related curriculum provided by teachers has a profound influence on children’s learning and development. Based on the research gaps discussed above, this study aims to gain an in-depth understanding of how ECE teachers have experienced professional learning with regard to ICT-related curriculum.

### TPACK model as a professional development framework

Teacher understanding of technologies is fundamental to their ICT implementation (Blackwell et al., 2016; Johnston et al., 2020). Some researchers have also argued that teacher understanding of pedagogies is critical, because it determines how teachers implement ICT in pedagogical activities (Nikolopoulou and Gialamas, 2015; Mertala, 2019; Vidal-Hall et al., 2020). These different understandings of the foundation of ICT implementation have resulted in multiple foci in relation to ICT-related professional development. As discussed above, some professional learning opportunities focus on the technical level while others emphasize teachers’ reflections on the pedagogical level of ICT use. This study believes that teacher understanding of technologies and pedagogies are both important for their ICT implementation; therefore, this study adopts the Technological Pedagogical Content Knowledge (TPACK) model (Mishra and Koehler, 2006) as its theoretical framework.

The TPACK model is a framework for investigating teachers’ instructional knowledge within the contemporary digital age (Mishra and Koehler, 2006). The foundation of the TPACK model is Shulman (1986) Pedagogical Content Knowledge (PCK), which has been regarded as a measure of the development of a teacher’s knowledge regarding how to design specific pedagogical activities (Pedagogical Knowledge) that enhance the teaching of subject-related content (Content Knowledge). With the arrival of technologies in educational settings, Technological Knowledge (TK, a teacher’s technology-related competence and skills) was regarded as another knowledge base by Mishra and Koehler (2006) to understand technology-supported teaching. There are interactions between different knowledge bases; Technological Pedagogical Knowledge (TPK) is the knowledge of the ways in which specific pedagogical practice can be facilitated by technology, and Technological Content Knowledge (TCK) is the understanding of how the subject matter can be combined with technology use. The TPACK model, therefore, refers to the repository of teacher understanding of how to use technology for specific pedagogical activities that enhance the teaching of specific subject matter (Mishra and Koehler, 2006).

The TPACK model has been widely regarded as a professional development framework for facilitating pedagogical practices with ICT. For example, researchers have introduced TPACK concepts to teachers, supported their pedagogical change (Koh, 2019), and used it as a model of professional learning to plan training that focuses on particular knowledge components (Angeli et al., 2016).

However, the relationships between TPACK components have been a debate among researchers, such as whether TPACK is a unique body of knowledge that helps to realize the transformation of knowledge or the integration of six single knowledge components (Angeli et al., 2016). This debate is significant because the actual relationships among knowledge components could explain whether training in relation to technology competence could effectively contribute to teachers’ TPACK development.

Given that the TPACK model has the analytical power to unpack the complicated stories behind teachers’ practices regarding ICT implementation (Park, 2015; Blackwell et al., 2016), this study adopts it as the theoretical framework for investigating Chinese ECE teachers’ professional learning experiences, by using it to guide data collection and the interpretation of the findings this will be explained in section: The current study.

## The context of China

In the context of globalization, educational authorities in China have introduced educational ideas from other countries (such as cognitive constructionism by Piaget and social constructionism by Vygotsky) into China in recent decades. Correspondingly, the educational principles adopted in kindergartens have evolved from encouraging teacher-directed teaching strategies to promoting child-centered teaching strategies, and from emphasizing collectivism to paying attention to individuality (Rao et al., 2010). The education reform puts forward many new requirements for kindergarten teachers, such as a kindergarten-base curriculum, pedagogical strategies, the ability to observe children and reflect on teaching (Jiang et al., 2017), and competence in using ICT for teaching (Dong, 2014; Luo et al., 2021a). This section discusses the common professional learning opportunities for kindergarten teachers in China and explains why investigating their ICT-related professional learning is important.

### Professional learning opportunities in China

In China, *teaching-research activities*/教研活动 (Song et al., 2014) is a main approach to support teachers’ curriculum-based professional learning. For an instance, the Jiangsu Provincial Department of Education (2017) indicates that to facilitate teachers’ abilities for observing children’s learning, teachers should conduct research on their own teaching. A common strategy for the teaching-research activities is *instructional viewing and emulating*/教学观摩. Kindergarten teachers participate in instructional viewing and emulating to construct pedagogical knowledge and transform practice through learning from peers, reflecting on instruction and receiving feedback from others (Huang et al., 2019).

In China, teachers usually need to participate in “lesson polishing”/磨课 (Yang and Li, 2017, p. 15) to prepare for instructional viewing and emulating. This implies that they practice giving the same lesson several times with similar content and presentation. In this way, teachers who participate in lesson-polishing can ask for comments from their colleagues and other people (such as principals) to adapt his/her teaching based on the feedback received (Yang and Li, 2017). Despite the possibility that the activity can benefit teachers by fostering peer learning, exercising reflection skills, and gaining access to collective wisdom, this article argues that it might be less beneficial for children because they experience similar whole-class activities repeatedly (if a teacher does “lesson polishing” with the same classroom).

Aside from the above-mentioned teaching-research activities, researchers have reported that some Chinese educational authorities often organize teaching skill competitions (either at the local level or at the national level) and use awards as a means of encouraging teachers to improve their teaching skills (Nong and Zhao, 2012; Song et al., 2014; Lu and Liang, 2016). Zhang and Ng (2011) investigated teacher appraisals in secondary schools in Shanghai and found that teachers’ rationales for participation are related to personal growth and honor; the honor could be given both personally and at the school level since individual winners can get awards, and their schools can gain the reputation and glory. By interpreting this phenomenon through Chinese traditional culture (i.e., collectivist ideology), Zhang and Ng (2011) indicated that teachers who participate in competitions are expected to work as a member of the collaborative group who can make contributions by bringing honor to the group.

This section has briefly introduced Chinese teachers’ professional learning opportunities, particularly the teaching-research activity. Those activities should be interpreted within the Chinese context; for example, a few possible reasons for lesson-polishing include the Confucian culture, which emphasizes self-improvement and drilling (Rao et al., 2010), and collectivist ideology, which stresses the power of collective actions (Zhu, 2011). These professional learning opportunities may have important roles to play in influencing ICT implementation in the ECE curriculum, but there has been little empirical evidence at present.

### ICT implementation in ECE contexts in China

The Ministry of Education of China (2012a) issued *10-Year Development Plan for Education Informatization (2011–2020)* and listed the construction of an ICT-supported learning environment within all educational contexts (including ECE) as a national development goal. Teachers’ integration of ICT in teaching and student-centered pedagogies as a means of catering to students’ different learning needs and interests is also encouraged by the plan. In the past decade, educational institutions at different levels (including kindergartens) have invested in their ICT infrastructure in terms of the amounts and types of devices (Luo et al., 2021a). Additionally, there have been new requirements for teachers’ ICT competence and understanding of how to use ICT to transform pedagogies.

The new requirements are revealed in some national documents. For example, the *Professional Standards for Kindergarten Teachers (Trial Version)* (Ministry of Education of China, 2011a) requires kindergarten teachers to possess a broad range of knowledge and skills, including knowledge related to ICT: “基本要求: 具有一定的现代信息技术知识。[Basic requirement for kindergarten teachers: Have enough knowledge about modern information technology.]” (Ministry of Education of China, 2011a, p. 13). In another document, *Teacher Education Curriculum Standard (Trial Version)* (Ministry of Education of China, 2011b), pre-service kindergarten teachers are expected to attend a course focusing on the application of modern educational technologies. These evidence demonstrate that kindergarten teachers are expected to gain ICT skills for teaching. However, those requirements are not always explicit and teachers may interpret questions like “what type of ICT implementation is expected” and “how ICT should be used in teaching” in different ways, which would further shape their actual implementation practices.

Few researchers have reported the current state of teachers’ professional learning in relation to ICT implementation in kindergartens in China, noting that the teachers have generally received technology skills training (Dong, 2014; Liu, 2017) while training related to how to use ICT for better pedagogies is lacking. By reviewing several empirical studies on the current situation of ICT implementation in ECE in China, Luo et al. (2021b) have argued that ICT-related training should meet teacher demands and be relevant to the pedagogical aspect of ICT implementation. Previous researchers have attributed the inappropriate use of ICT to a lack of training opportunities (Dong, 2014; Liu, 2017). Given that the training opportunities may influence how teachers combine ICT with the kindergarten curriculum and then profoundly influence children’s development, it is important to gain an in-depth understanding of the current state of ICT-related professional development in the ECE context in China.

## The current study

### Research design

Chinese educational authorities have made efforts to support teachers’ learning of advocated educational ideas by supervising teaching-research activities and organizing teacher competitions. However, researchers have argued that many teachers are still struggling to put those ideas into practice (Jiang et al., 2017). This issue may also happen to their practices about ICT implementation and relevant curriculum. Although the implementation of ICT in teaching and learning has been listed in China’s national development guidelines, little has been reported about how kindergarten teachers are supported in learning about ICT implementation. Therefore, this article aims to address the following research question: In what ways have teachers experienced professional learning opportunities in relation to ICT implementation in kindergartens in China? Drawing on the TPACK model, the findings can also demonstrate in what ways these ICT-related professional learning activities influence teachers’ ICT implementation in teaching practices.

This article reports on part of a doctoral research project that explored kindergarten teachers’ ICT-related perceptions, practices and professional learning experiences (Yang, 2021). The study is based on interpretivism (a research paradigm that believes multiple realities exist and are relative to individuals who have “particular sense-making, constructions, or meanings’, see Lincoln and Guba, 2013, p. 46) and it adopts a qualitative research design to gain an in-depth understanding of how teachers interpret and ascribe meaning to their experiences relating to ICT implementation. This article discusses teacher participants’ ICT-related professional learning experiences in particular.

The research design of the study draws on the theoretical framework, the TPACK model. This is because the TPACK model emphasizes individual teachers’ personal experiences and their own construction of knowledge related to technology, pedagogy, and content (Mishra and Koehler, 2006). Based on the research objective, it is important for the current study to consider teachers’ TPACK when investigating their personal interpretations of, and experiences related to, ICT-related training. Therefore, the interpretive qualitative research design was adopted to investigate teachers’ perceptions and experiences in-depth.

### Contexts and participants

The setting of this research is located in an eastern city with well-developed educational resources and socio-economically advantaged development (Ministry of Education of China, 2012b). To select information-rich cases to address the research questions (Patton, 2015), this study purposefully selected kindergartens that are equipped with various types of ICT devices. Based on different types and ranks, three kindergartens were selected: River Kindergarten is government-funded, Lake Kindergarten is university-funded, and Stream Kindergarten is self-financed. Furthermore, River Kindergarten is an ICT-exemplary kindergarten, which means it has a reputation in terms of an ICT-supported curriculum. Furthermore, this study purposefully selected teachers based on two criteria: they should have at least 10 year of experience in implementing ICT, and they should differ in terms of ages, educational backgrounds, teaching experiences and qualifications. Fifteen teachers were recruited for the study.

Among the participants, two were from the Lower Class (serving children aged 3–4 years old), five were from the Middle Class (serving children aged 4–5 years old) and eight were from the Upper Class (serving children aged 5–6 years old). Nine participants were in their 20s, with 1 to 5 years of teaching experience; the rest of the participants were in their 30s, with generally over 10 years of teaching experience. For educational backgrounds, 11 participants had a bachelor’s degree and four participants had vocational college diplomas. All participants gave informed consent for the research. In this article, the names of teachers and kindergartens are pseudonyms used to enhance confidentiality. This study has gone through strict ethical review by the University of Auckland Human Participants Ethics Committee.

### Data collection and analysis

To answer the research question, this study needs to understand how teachers have experienced professional learning in relation to ICT implementation and how they ascribe meanings to those experiences. Therefore, individual interview was adopted for its value in accessing teacher perception and experience. The interview protocol focuses on questions such as what types of training they had received in relation to ICT implementation and in what ways they thought the training was valuable or not. Based on the TPACK model, the interview protocol also focused on questions regarding technological and pedagogical aspects of participants’ perceptions and training experiences. The format of individual interview was semi-structured, which allowed the researcher to further investigate the meaning behind participants’ narratives. The researcher often prompted participants’ responses related to TPACK components (such as in what ways their technological pedagogical knowledge was developed *via* a professional learning activity) to address the research question. With the participants’ permission, all interviews were recorded for data analysis.

Document analysis was another research method used in the investigation, as it can help the researcher gain contextual information and develop topic-related knowledge (Merriam, 1998). In this study, public documents and private documents were included in the analysis to understand the research topic. In particular, public documents included national documents and guidelines, and websites of national or local competitions among teachers, such as *Professional Standards for Kindergarten Teachers (Trial Version)* (Ministry of Education of China, 2011a) and *Teacher Education Curriculum Standard (Trial Version)* (Ministry of Education of China, 2011b). This type of document can be valuable for investigating educational authorities’ expectations for teachers’ competence and professional learning, including those regarding ICT implementation. Meanwhile, teachers’ personal documents, such as teaching plans for instructional viewing and emulating and training notes, were collected to further understand their actual practice and training experiences. More importantly, the document analysis also offered an opportunity to triangulate the interview data (Patton, 2015); the alignments and misalignments between documents and teacher narratives can reflect what the policy-practice gap looks like, and the reasons for the gap can shed light on the analysis of data.

The study adopted thematic analysis to investigate patterns and themes in collected information (Patton, 2015). The data analysis included the following steps. First, the researcher transcribed and translated (from Chinese to English) all interview recordings and read and re-read collected documents for familiarity. Then, the researcher searched for recurring elements and analyzed the transcripts and documents both inductively (i.e., keeping an open mind for frequent units that appeared in the data) and deductively (i.e., being influenced by some pre-determined topics, such as TPACK components). Next, the researcher considered how different codes would combine to form a theme or sub-theme. For example, the codes “learning new approaches to implementing ICT” and “seeing the potentials of ICT use” were combined into a sub-theme: “The perceived benefits of visiting other kindergartens.” After doing so, the researcher continued to investigate the relationships between themes and sub-themes. For instance, the sub-themes “the perceived benefits of visiting other kindergartens” and “teachers” preparation for holding visits’ worked together in telling a holistic story related to the theme “visits as a professional learning approach.”

In addition to the inductive analysis approach, this study also adopted a deductive method by using the TPACK model to guide this process. For example, the researcher intentionally searched for participants’ TK, PK, TPK, and TCK in their narratives about ICT implementation and previous training. These knowledge bases were developed into various themes, working with other themes to tell a whole story about participants’ training experiences. The relationships between different knowledge components were also identified (for example, the connections between TK, PK and TPK), which helped the researcher group and organize the sub-themes and themes. This process can also shed light on how professional learning on a single knowledge base could contribute to overall ICT implementation. In summary, the interpretation of interview data was based on the meanings of themes as well as the connections between themes and sub-themes.

The analysis of collected documents was mainly based on a deductive approach, as the researcher purposefully searched for information about the macro-level requirement or expectations related to teachers’ ICT implementation, ICT competence, and professional learning. These aspects were included in the analytical category to examine the data and address the research question.

## Findings and discussion

This study found participants have received diverse types of professional learning opportunities in relation to ICT implementation, including kindergarten-based training, professional learning opportunities provided by the local education bureau and other teaching-research activities (e.g., *lessons for viewing*/剬开课and *visiting other kindergartens*/参观). Three themes emerged from participants’ narratives regarding ICT-related professional development and collected documents: *ICT-related training and technical support; visits and lessons for viewing*; and *competitions for teachers*.

### ICT-related training and technical support

The analysis found that participants have experienced three types of ICT-related training, which were developed as three sub-themes for explaining the collected data. The three types are: *pre-and in-service training that focused on ICT-operating skills*, *training provided by the local school district which involved teachers from different educational contexts*, and *technical training organized by kindergartens*.

Firstly, this study found that ten teachers had attended compulsory courses on ICT during pre-service education, which helped them to learn how to use computers. Five teachers noted that they had not experienced ICT-related courses until they began to work in kindergartens. Tong (30 years old, Lake Kindergarten) described the courses meant to deliver knowledge and skills regarding common software, such as how to use PowerPoint to make animations. When asked about the value and usefulness of the courses, Xie (24 years old, Lake Kindergarten) and the other two participants indicated that “I learned something from it, because I did not know those functions before”; however, Xie also indicated that the courses might not be necessarily useful for her pedagogy because she did not find the opportunity to combine those functions with her teaching. Based on Xie’s narratives, the content of those courses does not always match its actual value in teaching practice. The review of collected documents (*Teacher Education Curriculum Standard* in particular) found that the description of the learning content for kindergarten teachers’ ICT implementation is extremely vague, with no explicit explanation or requirement regarding in what ways teachers should implement ICT in practice.

In addition to courses, some participants have also joined an “ICT research team’ organized by their local school district to learn ICT-relevant knowledge and skills. Dang (35 years old, Stream Kindergarten) indicated that members of the district-level research team, who came from different educational contexts (such as kindergartens and primary schools), had opportunities to discuss and share experiences in relation to how to adopt ICT as a teaching tool. Dang emphasized that she had learned from a primary school teacher how to make PowerPoint slides, such as how to insert pictures and videos. However, as previous researchers have noted (such as Dong, 2014; Ihmeideh and Al-Maadadi, 2018; Luo et al., 2021a), ICT implementation is highly contextualized, and this study argues that primary school teachers’ knowledge about how to use ICT in teaching might not be appropriate for kindergarten teachers because of children’s different age groups and different learning needs. The findings demonstrate that, although a teaching-research team is believed useful for teachers to develop teaching skills and improve practice through exchanging experience and collaborating (Wang, 2015; Jiangsu Provincial Department of Education, 2017), the value of such professional development is dependent on whether teachers’ knowledge and experiences can be appropriately and effectively transferred.

The third type of professional development identified in participants’ narratives was kindergarten-based training. This kind of training was often provided when new ICT devices arrived at kindergartens. Eight participants described this type of training as “not frequent,” indicating that it was mainly about “how to use the new hardware/software” with no instruction about “how to combine it with pedagogical activities” (Jing, 33 years old, River Kindergarten). In River Kindergarten, participants reported that the training sessions were organized based on the collaboration between kindergarten and ICT companies. Once the ICT device was introduced to the kindergarten and collaboration was established, the ICT companies would “set up their products in the kindergarten” and provide a training session on “how to use it” (Wan, 32 years old, River Kindergarten). Participants in the Stream Kindergarten also reported demonstration-focused training:

When the interactive whiteboard was just implemented, the principals invited someone outside the kindergarten to teach us how to use this device. However, he only told us how to turn on and turn off the device. (Fan, 37 years old, Stream Kindergarten)

It seemed that this type of training was generally a “one-off experience” as they did not have following communications with the staff. Fan emphasized that “training is just training; how to use it [ICT] is all about teachers’ decisions”; she explained that her ICT implementation was based on the teaching-and-learning goals. Given that ICT companies tended to provide one-off training with almost no attention to the pedagogical aspect, this study argues that this type of training might not directly contribute to appropriate ICT implementation. This finding is consistent with Vidal-Hall et al. (2020) and Luo et al. (2021a,b) who highlighted that training should be tailored to the needs of teachers and relative to the ECE pedagogies. More importantly, the importance of ongoing support for teachers’ ICT implementation has been noted by Sargent (2017), who argues it can bring about sustainable changes in knowledge and skills. However, this feature was not found in participants’ previous professional learning.

Analyzing based on the TPACK model, this study found that the technical training merely contributed to participants’ TK rather than other knowledge components (such as TPK). The above-mentioned Fan’s emphasis on the relationship between ICT implementation and the teaching-and-learning goals reflects that TPK should be a key focus during professional development to effectively prepare teachers for teaching in practice. In fact, nine teachers involved in this research believed that they had sufficient ICT competencies to implement ICT for daily teaching. Chinese kindergarten teachers generally utilized ICT as screen-based technologies in collective teaching (Dong, 2014; Liu, 2017) and this study argues that “sufficient” competence would not automatically contribute to their understanding of appropriate ICT implementation, for example, to support children’s spontaneous exploration. Thus, this study argues that teacher knowledge about using ICT as a tool to support child-centric pedagogies should be an important part of their professional development. Previous researchers (e.g., Blackwell et al., 2016; Sargent, 2017) have identified a lack of training as the main barrier preventing teachers from implementing ICT effectively. However, this study found that participants had received ICT-related technical training from various organizations including kindergartens, ICT companies and local school districts. This is inconsistent with previous research such as Dong (2014) and Liu (2017), which found ICT-related training opportunities for Chinese kindergarten teachers were very limited. This mismatch perhaps implies that, Chinese kindergarten teachers’ ICT-related training has received more attention in recent years due to educational authorities’ increasing attention toward the development of educational technologies (see Luo et al., 2021a,b) Nevertheless, this study found that kindergarten teachers’ ICT-related training was insufficient in terms of depth and scope because it tended to emphasize more on the technical level rather than the pedagogical level of ICT implementation. As previous researchers noted, understanding how to run a software program would not guarantee children’s meaningful learning with ICT (Sargent, 2017; Mertala, 2019). To better enhance teachers’ competence and skills for ICT implementation, more curriculum-based professional learning opportunities are needed.

### Visits and lessons for viewing

The second theme that emerged from participants’ narratives and collected documents is *visits and lessons for viewing*. This study found that *instructional viewing and emulating*/教学观摩 was an approach used for teachers’ curriculum-based professional learning, as participants had visited various kindergartens to learn about ICT implementation. This section discusses the findings based on two sub-themes: *how teachers perceive the values of visits and lessons for viewing*, and, *how teachers prepared for visits and lessons for viewing*, which work together in telling the story about participants’ professional development from two distinguished perspectives.

How teachers perceive the values of visits and lessons for viewing reflects a complicated story. For example, Tong held a positive attitude toward *visit*/参观, a common means of instructional viewing and emulating activities, indicating she “can truly learn many things” through observing how other teachers implemented it in various subject-based activities. Another nine participants in the research shared this viewpoint. Tong, on the other hand, stated that while she “learned” about the potential of ICT through visits, she did not put them into daily practice because of the belief that ICT implementation should be based on kindergartens’ own curricula. This conclusion resonates with a study in Shenzhen (Huang et al., 2019), where researchers believed this form of professional learning was “low-quality” due to the disconnect between visits (i.e., viewing) and practice. Given the lack of attention on the pedagogical level, the present study contends that these activities are not practical enough for teachers. More practical guidelines and support, according to Huang et al. (2019), are needed to ensure the value of this type of professional learning.

Through a TPACK lens, the reasons why this form of professional learning is “low-quality” is two-folded. Firstly, while teachers’ observation of others’ ICT implementation is useful for developing their understanding of ICT, their TPACK might not be developed due to the lack of reflection. Vidal-Hall et al. (2020) have noted that teachers’ observation and reflection are equally important because they work together in contributing to the modifications of perception and practice. TPACK has a heavy reliance on personal construction of different knowledge components and experiences of teaching in practice (Angeli et al., 2016; Koh, 2019), and therefore this type of professional learning might not develop teachers’ TPACK to transform practice. More importantly, teachers’ TPACK is highly contextualized and their practice is bound to kindergarten curricula, children’s learning needs, and kindergarten-level cultures (Blackwell et al., 2016). Teachers’ TPACK might not be smoothly transferred without consideration of these contextual factors. As for the second sub-theme, the other side of the story related to visits can be revealed from information gathered from River Kindergarten, an ICT-exemplary kindergarten. River Kindergarten often organized visits to allow teachers from other kindergartens to view and emulate ICT implementation. However, according to Lun (36 years old, River Kindergarten), the preparation phase included various tasks, such as cleaning, charging devices in advance, decorating the environment, and preparing *a lesson for viewing*/剬开课. Those “lessons,” as the kindergarten website shows, were generally about whole-class activities involving ICT-supported instruction. Xuan explained the preparation work for an ICT-supported lesson:

For a 剬开课 [the lesson for viewing], we need to write a teaching plan carefully, and practice it with children several times. Through conducting the teaching again and again, we can reflect on our practices and get feedback from experienced teachers in this kindergarten. Only in this way could we ensure a good result. (Xuan, 27 years old, River Kindergarten)

Xuan mentioned *lesson-polishing*/磨课 in describing her preparation, which is a common professional development approach used in diverse educational settings in China (Zhu, 2011; Yang and Li, 2017). However, as this practice means that similar teaching activities may happen several times, children may gain “repeated” or similar learning experiences by receiving fewer opportunities for active exploration. Therefore, this study argues that this format of professional learning is not appropriate for kindergartens because it works for the benefit of teachers rather than of the children. This reflects a long-held Chinese educational belief that teachers have a dominating position in educational settings because they possess the expertise required for knowledge-delivering (Rao et al., 2010). The findings reported in this section concern how teachers have experienced ICT-related visits as part of their professional development. The review of River Kindergarten’s website found that the responsibilities of ICT-exemplary kindergarten include providing guidance to other kindergartens and promoting pedagogical interactions among kindergartens. Therefore, visits were described on the website as “teaching-research activities” aimed at promoting ICT implementation in other kindergartens. However, the value of this form of “teaching-research activities” needs further analysis and the data reflect a policy-practice gap. The analysis of collected documents found that Jiangsu Provincial Department of Education (2017) has provided suggestions about kindergarten teaching-research activities:

… teachers should not sacrifice children’s development, should not lead children to have activities in a performance format, should not train a group of children for their research work;

… teachers should not organize research work which is out of children’s playful context and out of kindergarten’s daily activities … (p. 2)

The key point of this document is that teaching-research activities should not disrupt kindergarten normal activities. While Lun claimed that “it did not interrupt us because it’s for the kindergarten development’, the analysis of findings reflected the disruptions clearly. Being consistent with research in other education contexts in China (Wang, 2015; Lu and Liang, 2016), this study found that this type of professional development might influence children’s development (by taking them out of the daily learning context), consume teachers’ time and energy, and not always benefit visitors’ practices. The suggestion from the study aligns with previous researcher’s argument that “few teaching-research activities involve reflections about the actual value of these activities, [and] some [teaching-research] activities are not necessary but are conducted widely” (Wang, 2015, p. 62), calling for critical reflections on the value of teaching-research activities. These problems identified in the study, perhaps, are the reason why the Jiangsu Provincial Department of Education issued guidelines for kindergarten teaching-research activities.

### Competitions for teachers

As competitions among teachers have been considered an effective approach to assist professional development in China (Nong and Zhao, 2012; Lu and Liang, 2016), the ICT-related competition was the third theme found in participant narratives and collected documents. Five participants mentioned ICT competitions held by educational authorities when asked whether they felt being encouraged to implement ICT in teaching; Wan (32 years old, River Kindergarten) strongly argued, “the competition itself is an encouragement, is not it?’ It appeared that the general educational context has shaped the ECE context, making participants regard ICT implementation as an expectation of the educational authorities.

The competitions mentioned by participants included *Nanjing ICT-Supported Teaching Master Competition* (teaching master competition hereafter) and *Nanjing Micro-lesson Competition for Teachers in Primary and Secondary Schools* (micro-lesson competition hereafter). Those competitions required each participant to make a video of one lesson and send the recording to the local education bureau for evaluation. The evaluation criteria for the teaching master competition were collected (Nanjing Education Bureau, 2018), including: the design of ICT-supported teaching activities, teachers’ proficiency with ICT skills, whether the implementation of ICT can effectively support teaching, and whether the effect of ICT-supported teaching is distinct from “traditional teaching.” These criteria demonstrate that the aim of this competition is to encourage teachers to combine ICT with teaching appropriately. Through a TPACK lens, these competitions seem like an examination of teachers’ TPACK and its application in teaching practice, however, this study argues that the competition may not holistically reflect teachers’ TPACK because the teaching activity is short (explained later) and it does not happen in real kindergarten context.

This paper specifically discusses teacher experiences related to the micro-lesson competition because twelve participants were preparing for it during the data collection period. The collected documents (i.e., website for the micro-lesson competition) showed that this competition is also open to kindergarten teachers despite its title (i.e., for teachers in primary and secondary schools). The website also indicated that a micro-lesson is “a video-recording of a short, but complete teaching activity based on specific teaching content” (Nanjing Education Bureau, 2017, p. 2) and competition aimed at “improving teachers” pedagogical planning ability, contributing to the sharing of micro-lesson resources, and accelerating the reform of pedagogy’ (p. 2). The micro-lesson competition targeted not only ICT-supported teaching but also wider teaching activities, and its aim that “accelerating the reform of pedagogy’ can be supported by the implementation of ICT; thus, this study adopts the micro-lesson competition as an instance of three issues surrounding teacher competition.

The first issue is the disconnect between the micro-lessons designed only for the competition and teachers’ normal practice. Qiong (24 years old, Lake Kindergarten) indicated that the aim of making micro-lesson recordings was merely “being involved in this competition.” Qiong had played the micro-lesson recordings for children, but later realized that “children would not benefit from watching tutorial videos about folding papers; I think teachers’ direct instruction is better” and began to doubt the educational value of those recordings. Qiong deduced that perhaps it [the micro-lesson] could benefit teachers if the recordings could be uploaded to a website for sharing with other teachers’. Qiong’s assertion mirrored the data from document analysis, as the aim mentioned on the competition website is that the micro-lesson recordings are valuable resources for teachers to learn from others and reflect on their practices. Nevertheless, like other teaching-research activities, the aim of the competition was relative to teacher professional development (e.g., teaching competence and skills) rather than the development of the children. Thus, this study argues that while developing teachers’ TPACK is important, it should not be aimed as an end in itself. The ultimate goal for developing teachers’ TPACK should be closely related to children’s learning and development.

The second issue is related to the amount of time needed to prepare for the competition. Wan indicated that she had to “plan the activity carefully, mock the activities several times, and edit the recordings’ and therefore needed to spend much time on it. The review of the competition website also found that video-recordings should last for five to eight minutes and contain captions about the main steps of the activity. This procedure seems time-intensive. Participants’ narratives reflected that while the competition was designed to encourage the sharing of educational materials to improve teaching abilities, it was time-consuming and ineffective in fact. Wan’s narratives reflected that, again, participating in the competition requires teachers to repeatedly “mock” teaching activities, which may decrease children’s opportunities for free play and spontaneous explorations.

The third issue that emerged from participants’ narratives is about their choices in relation to competition participation. While Qiong and Wan doubted the value of micro-lessons, neither of them said no to the participation because they believed that “the principal hoped for our active engagement.” While participants all used the word “voluntary” to describe their choice, they later noted they were “nominated” by principals and they hoped to meet principals’ expectations. This shows that participation in ICT-related competitions was driven by top-down decisions and was disconnected from children’s actual learning needs. This finding aligns with previous researchers’ argument that structural hierarchies and making efforts to bring honor to a collective group are rooted in Chinese traditional culture (Zhang and Ng, 2011). In other education contexts such as secondary school education, it has been found that these beliefs have shaped the school culture in various ways (such as emphasizing individual teacher growth and contribution, see Zhang and Ng, 2011; Song et al., 2014). This phenomenon has also been found in the current study, which is in the context of kindergarten education. Being profoundly shaped by the social and cultural traditions, teachers’ perceptions of competition and emphasis on group honor further influenced their choices and practices, according to the findings.

This section has reported how teacher competition, as a professional development approach, influenced teachers’ ICT-related practices. Based on the analysis of three issues regarding this professional development approach, this study argues that the competitions could occupy teachers’ time and prevent them from investing time in planning meaningful activities. There has been no research into how teacher competition facilitates the development of TK, PK, and TPK, thus the usefulness of this form of professional development is still unknown. Additionally, the tensions experienced by participants deserve further investigation because they may also exist in other competitions, such as the ICT-supported teaching master competition. Based on teachers’ voices identified in this study, the following section discusses implications for practice, policy-making, and future research directions.

## Conclusion

This qualitative study investigated the experiences of a group of teachers’ professional learning that focuses on the ICT-related curriculum in China. It was found that participants had received various professional learning opportunities related to ICT implementation and relevant curriculum. The analysis shows that although these opportunities were offered by different organizations, they all paid much attention to the technical level of ICT use. Following previous research (Dong, 2014; Blackwell et al., 2016), this study argues that the narrow focus on professional development would not guarantee better ICT implementation. The findings also show that some professional learning opportunities were for the teachers’ sake rather than the children’s sake, as organizers prioritized professional learning and practice while paying less attention to the influences on children’s learning and development. In line with some previous studies in China (Song et al., 2014; Huang et al., 2019), this study found that some professional learning opportunities did not occur in the classroom-based format, which would cause a gap between professional learning and teaching practice and even consume teachers’ time and energy.

This study has strengths in developing an in-depth understanding of how a small group of kindergarten teachers have experienced ICT-related professional learning opportunities and providing rich descriptions of the contexts. Although this study was conducted in China and the findings should be understood with caution in relation to other contexts, it still makes significant contributions. First, considering the gap in this research topic, this study contributes to the current understanding of ICT-related professional development by discussing the complexities of different professional development activities. Second, the culture underpinning professional development (teacher competition in particular) in ECE in China, which seems viewing-oriented, has not been explicitly discussed in the literature. Therefore, this study provides empirical evidence for the current situation of professional development in China and an in-depth discussion about the influence of the social and cultural traditions on teachers’ professional learning. Third, this study shed light on theories concerning teacher knowledge components around the use of ICT, as the findings demonstrate that the development of a single knowledge base (for example, technological knowledge) would not result in the development of the entire body of TPACK. Thus, this finding adds more evidence about the complex interrelations between TPACK components. Researchers have suggested that teachers need to transform their PK to TPK for ICT implementation (Nikolopoulou and Gialamas, 2015; Angeli et al., 2016; Blackwell et al., 2016) while this study argues that teachers may struggle with the transformation without appropriate professional learning opportunities.

The findings of the study make some implications for teachers, principals, policy-makers and researchers in terms of practices, professional development programs, and future research directions. First, this study follows previous researchers’ recommendations (Gibbons, 2010; Vidal-Hall et al., 2020), arguing that more practice-oriented professional learning is needed for supporting teachers’ ICT implementation in the kindergarten curriculum. A learning community that involves curriculum-based and classroom-based learning opportunities, teachers’ active participation, peer discussion, and critical reflection could be an approach to ICT-related professional development (Johnston et al., 2020). These professional learning opportunities can help teachers engage in observation and critical reflections on current ICT implementation to bring about change in practice (Vidal-Hall et al., 2020). Considering the narrow focus of professional learning identified in the study as well as the child-centric pedagogies advocated in recent education reform in China, it is also important to ensure early childhood teacher education and professional development program to provide ongoing support for teachers to go beyond the “operating” aspect of ICT implementation, and to develop knowledge about ICT use for supporting children’s free play and self-initiated activities (i.e., technological pedagogical knowledge). This would guarantee teachers implement ICT based on curriculum content, pedagogical purposes, and children’s actual needs and interests (Yang, 2022).

Second, the findings imply that principals and policy-makers need to evaluate the benefits of teaching-research activity and its influence on teachers’ practice as well as children’s learning. As researchers have argued that the environment (school culture, in particular, see Zhang and Ng, 2011) profoundly shapes teacher development, this study calls for changes in the top-down decision-making process about teachers’ participation in professional development. It is necessary for principals to empower teachers to make their own decisions about whether and in what ways to participate in professional development (such as teacher competitions). Dialogues between teachers and principals to create a shared understanding in relation to ICT implementation (Johnston et al., 2020) and professional learning (Song et al., 2014) are also needed. More importantly, principals should re-think the potentially negative influences of professional development (such as lesson-polishing) on children’s development and policy-makers should prioritize this aspect when designing professional development programs.

Some issues about teachers’ ICT-related professional learning have been found in the three kindergartens under the study, but have not been addressed in this article. Therefore, there is a need to conduct further investigation into research questions such as what types of professional development can support teachers to implement ICT appropriately. Based on the issues about teacher competitions found in the study, future researchers can also examine in what ways this professional development approach facilitates teachers’ teaching skills and ICT competencies. Investigation into these aspects will then provide more empirical evidence for stakeholders and assist them to modify professional development approaches to facilitate teachers’ practice.

## Data availability statement

The raw data supporting the conclusions of this article will be made available by the authors, without undue reservation.

## Ethics statement

The studies involving human participants were reviewed and approved by University of Auckland Human Participants Ethics Committee. The patients/participants provided their written informed consent to participate in this study.

## Author contributions

TY is mainly in charge of the research design, data collection, data analysis, and drafting the manuscript. XH is responsible for conception of the work and the revision of the manuscript. All authors contributed to the article and approved the submitted version.

## Funding

This work was funded by the International Joint Research Project of Faculty of Education, Beijing Normal University (Approval No. ICER202202) and Guangdong Provincial Education Science Program (grant number 2022GXJK421).

## Conflict of interest

The authors declare that the research was conducted in the absence of any commercial or financial relationships that could be construed as a potential conflict of interest.

The reviewer XL declared a shared affiliation with the authors TY and XH to the handling editor at the time of review.

## Publisher’s note

All claims expressed in this article are solely those of the authors and do not necessarily represent those of their affiliated organizations, or those of the publisher, the editors and the reviewers. Any product that may be evaluated in this article, or claim that may be made by its manufacturer, is not guaranteed or endorsed by the publisher.
